# Pharmacological Mechanism of Danggui-Sini Formula for Intervertebral Disc Degeneration: A Network Pharmacology Study

**DOI:** 10.1155/2021/5165075

**Published:** 2021-11-11

**Authors:** Longjie Wang, Jialiang Lin, Weishi Li

**Affiliations:** ^1^Peking University Third Hospital, Department of Orthopaedics, Beijing, China; ^2^Engineering Research Center of Bone and Joint Precision Medicine, China; ^3^Beijing Key Laboratory of Spinal Disease Research, China

## Abstract

**Background:**

Intervertebral disc degeneration (IVDD) is the most significant cause of low back pain, the sixth-largest disease burden globally, and the leading cause of disability. This study is aimed at investigating the molecular biological mechanism of Danggui-Sini formula (DSF) mediated IVDD treatment.

**Methods:**

A potential gene set for DSF treatment of IVDD was identified through TCMSP, UniProt, and five disease gene databases. A protein interaction network of common targets between DSF and IVDD was established by using the STRING database. GO and KEGG enrichment analyses were performed using the R platform to discover the potential mechanism. Moreover, AutoDock Vina was used to verify molecular docking and calculate the binding energy.

**Results:**

A total of 119 active ingredients and 136 common genes were identified, including 10 core genes (AKT1, IL6, ALB, TNF, VEGFA, TP53, MAPK3, CASP3, JUN, and EGF). Enrichment analysis results showed that the therapeutic targets of DSF for diseases mainly focused on the AGE-RAGE signaling pathway involved in diabetic complications, IL-17 signaling pathway, TNF signaling pathway, Toll-like receptor signaling pathway, apoptosis, cellular senescence, PI3K-Akt signaling pathway, and FoxO signaling pathway. These biological processes are induced mainly in response to oxidative stress and reactive oxygen species and the regulation of apoptotic signaling pathways. Molecular docking showed that there was a stable affinity between the core genes and the key components.

**Conclusions:**

The combination of network pharmacology and molecular docking provides a practical way to analyze the molecular biological mechanism of DSF-mediated IVDD treatment, which confirms the “multicomponent, multitarget and multipathway” characteristics of DSF and provides an essential theoretical basis for clinical practice.

## 1. Introduction

Low back pain is the sixth largest disease burden globally and is the leading cause of disability, which has become a global health concern [1]. A previous study showed that 40% of low back pain is related to intervertebral disk degeneration (IVDD) [2]. The major pathological features of IVDD are the elevated expression of inflammatory mediators, increased senescence and apoptosis of nucleus pulposus cells (NPCs), and degradation of the extracellular matrix [3, 4]. Importantly, IVDD may bring about a range of clinical symptoms, such as pain, numbness, and even paralysis of the lower extremities, causing enormous suffering and economic burden.

The initial clinical treatment for IVDD has focused on symptom relief, mainly with nonsteroidal anti-inflammatory drugs (NSAIDs) [5, 6]. However, studies on the use of NSAIDs in articular cartilage have shown detrimental effects [7–9]. Therefore, there is an urgent need for an effective and safe drug. Traditional Chinese medicine (TCM) has been used to treat various diseases for thousands of years [10]. However, due to the diversity of TCM components and the complexity of the body, systematic research on TCM has been limited.

Network pharmacology is a strategy for elucidating the synergistic effects and potential mechanism of multicompound and multitarget drugs based on various complex “drug-ingredient-target gene” networks [11]. It may be a potential tool to systematically explore new applications for TCM. Recent studies demonstrated that network pharmacology could achieve high performance in the prediction of the mechanisms of TCM involved in treating diseases [12, 13]. We hypothesize that combining gene ontology (GO) and Kyoto Encyclopedia of Genes and Genomes (KEGG) analysis with network pharmacology may provide more valuable and complementary information, thereby further improving the prediction performance of potentially effective mechanisms.

The Danggui-Sini formula (DSF) is a classic prescription in the Treatise on Febrile Diseases and is mainly composed of seven herbs (Table 1): *Angelica sinensis* (Dang Gui, DG), *Cinnamomum cassia Presl* (Gui Zhi, GZ), *Cynanchum otophyllum Schneid* (Bai Shao, BS), *Asarum sieboldii Miq* (Xi Xin, XX), *Glycyrrhiza uralensis Fisch* (Gan Cao, GC), *Tetrapanax papyriferus* (Tong Cao, TC), and *Ziziphus jujuba Mill* (Da Zao, DZ). In recent years, pharmacological studies have shown that this decoction has positive anti-inflammatory and antioxidative effects, especially in the treatment of osteoarthritis [12, 13]. Although Chinese medicine has already used DSF to treat IVDD, its mechanism of action needs to be further studied [14].

**Figure 1:**
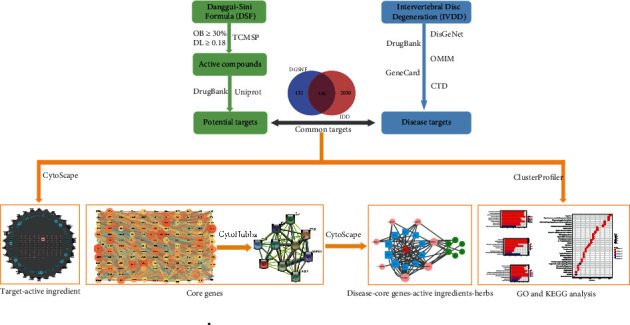
Flowchart to explore the possible mechanisms of the Danggui-Sini formula (DSF) for intervertebral disc degeneration (IVDD).

This study integrated network pharmacology and molecular docking to explore the potentially effective mechanism and targets of DSF acting on IVDD. The research process is shown in Figure 1. The results highlight the potential of DSF in the treatment of IVDD.

## 2. Materials and Methods

### 2.1. Active Ingredients and Target Genes of the Danggui-Sini Formula

By searching the Traditional Chinese Medicine Systems Pharmacology (TCMSP) database (https://tcmsp-e.com/) [15], with the filter conditions of oral bioavailability (OB) ≥ 30% and drug likeness (DL) ≥ 0.18, the active ingredients of each herb from DSF were acquired. At the same time, the corresponding target protein information of the active compounds was obtained through the DrugBank database (https://go.drugbank.com/) [16], and the conversion of gene symbols was completed through the UniProt database (https://www.UniProt.org) [17].

### 2.2. Related Targets of IVDD and Common Gene Set

To obtain the targets of IVDD, we searched a total of five databases, namely, Online Mendelian Inheritance in Man (OMIM) (https://omim.org/) [18], Genecards database (https://www.genecards.org/) [19], Comparative Toxicogenomics Database (CTD) (http://ctdbase.org/) [20], DrugBank database (https://go.drugbank.com/) [16], and DisGeNet database (https://www.disgenet.org/) [21]. In addition, all targets were standardized in the UniProt database [17]. A common gene set of “compound-targets-disease” comprises the potential target genes of DSF for IVDD and was built by creating a Venn diagram.

### 2.3. Network Visualization and Enrichment Analysis

“Active ingredients - potential targets” network and another related network for disease-core genes–active ingredients–herbs were established using the Cytoscape software [22]. To obtain more accurate gene annotation results, we utilized the clusterProfiler package of the R platform for gene ontology (GO) and Kyoto Encyclopedia of Genes and Genomes (KEGG) functional enrichment analysis [23]. GO enrichment analysis revealed the underlying mechanisms from biological processes (BPs), cell components (CCs), and molecular functions (MFs), while KEGG is a pathway-related database.

### 2.4. Protein–Protein Interaction (PPI) Network Construction and Core Gene Selection

We imported the common genes into the STRING database (https://www.string-db.org/) [24] to obtain the PPI network and TSV format file with the parameters of Organism = Homo sapiens and moderate confidence = 0.400. Then, TSV files were imported into the software to realize PPI network visualization and further analysis. The CytoHubba plug-in was used to identify the top 10 core genes by using 12 kinds of topological measures.

### 2.5. Molecular Docking

Molecular docking analysis focused on the proteins encoded by the 10 core genes and their corresponding active components. First, we downloaded the 3D structure of the protein from the RCSB PDB database (https://www.rcsb.org/) and used the PyMol 2.4.0 software to remove water and natural ligands from the protein. Second, the 2D structures of active components (small molecular ligands) were downloaded from the PubChem database (https://pubchem.ncbi.nlm.nih.gov/). Then, we used the ChemBio3D software to convert the molecular ligand into a 3D structure and performed energy minimization by the MM2 calculation method. Hydrogenation of proteins and small molecular ligands was prepared with the Autodock tool. Finally, Autodock Vina was used for molecular docking and calculating the binding energy [25].

## 3. Results

### 3.1. Active Ingredients in DSF and Potential Genes

Seven herbal medicines of the DSF were successively inputted into the TCMSP database, and a total of 155 active ingredients were obtained, among which 2 were from DG, 7 were from GZ, 13 were from BS, 8 were from XX, 92 were from GC, 4 were from TC, and 29 were from DZ (Supplementary Table 1). Then, we searched the DrugBank database for targets of each active ingredient and obtained a total of 268 genes after removing duplicates. All gene names were corrected through the UniProt database. Details of the above information are listed in Supplementary Table 2.

**Figure 2:**
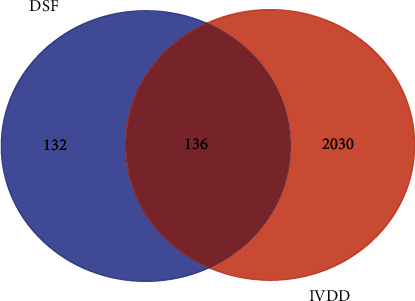
Venn diagram for DSF-related genes and IVDD-related genes.

With the keyword “intervertebral disc degeneration,” we searched the five databases mentioned above and obtained a total of 2166 genes after deduplication. Using an online web service (http://bioinformatics.psb.ugent.be/webtools/Venn/), we obtained common genes that intersect between drug-target and disease-related genes. One hundred thirty-six genes were identified as potential genes for DSF in the treatment of IVDD, and the Venn diagram is shown in Figure 2 and Supplementary Table 3.

### 3.2. Active Ingredient-Potential Target Network

**Figure 3:**
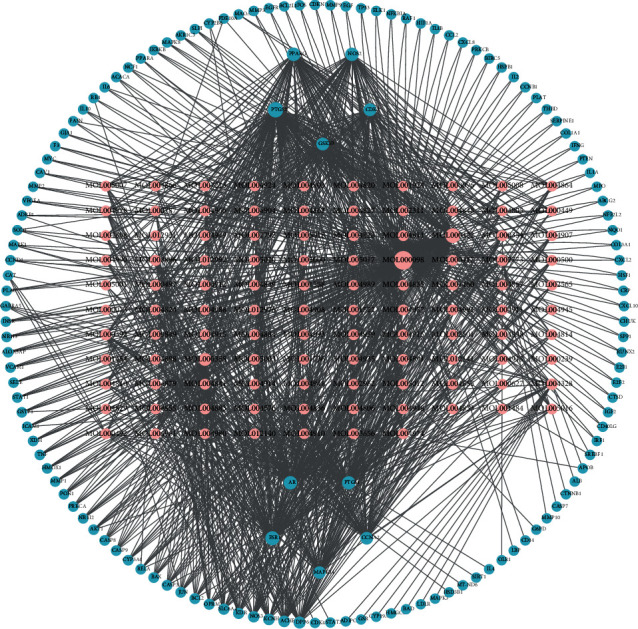
Construction of a common gene-active ingredient network. Blue nodes represent the common targets of IVDD and DSF; pink nodes represent the active ingredients related to the common targets. The line between two nodes represents the interaction; the size of each node represents the number of connections.

After obtaining the common genes and their corresponding active compounds, a network of active ingredient-potential targets was drawn by using Cytoscape (Figure 3). The network has 243 nodes and 1214 edges. Furthermore, we found that one gene corresponds to multiple active ingredients and vice versa. According to the number of target nodes, we ranked the active ingredients, with quercetin (MOL000098) targeting the most genes.

### 3.3. PPI Network and Core Genes

**Figure 4:**
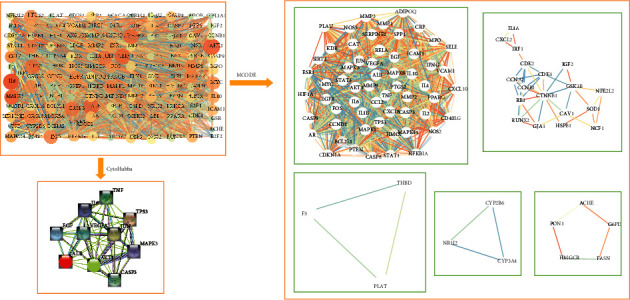
Protein–protein interaction (PPI) network and core genes: (a) the protein–protein interaction (PPI) network of potential targets of DSF in the treatment of IVDD; (b) PPI network of the core genes; (c) cluster analysis of the common targets.

To understand the relationship among common genes and obtain core genes, 136 common genes were first imported into the STRING database, and then, the PPI network and TSV format file were obtained (Figure 4(a)). Second, we visualized the PPI network by importing it into Cytoscape and identified the 10 core genes using Cytohubba plug-ins with 12 kinds of topological sorting. Finally, we found that the top 10 core genes obtained by the two methods of maximum neighborhood component (MNC) and degree were the same, namely, AKT1, IL6, ALB, TNF, VEGFA, TP53, MAPK3, CASP3, JUN, and EGF (Figure 4(b)). We performed further cluster analysis of the PPI network with the MCODE plug-in and acquired 5 clusters (Figure 4(c), Table 2). The top 1 cluster contained 10 core genes, which further confirmed the criticality of core genes.

### 3.4. GO and KEGG Analysis

To further explore the interaction between common target genes and the mechanism by which DSF may treat IVDD, GO functional analysis and KEGG pathway enrichment analysis were performed using the R platform.

**Figure 5:**
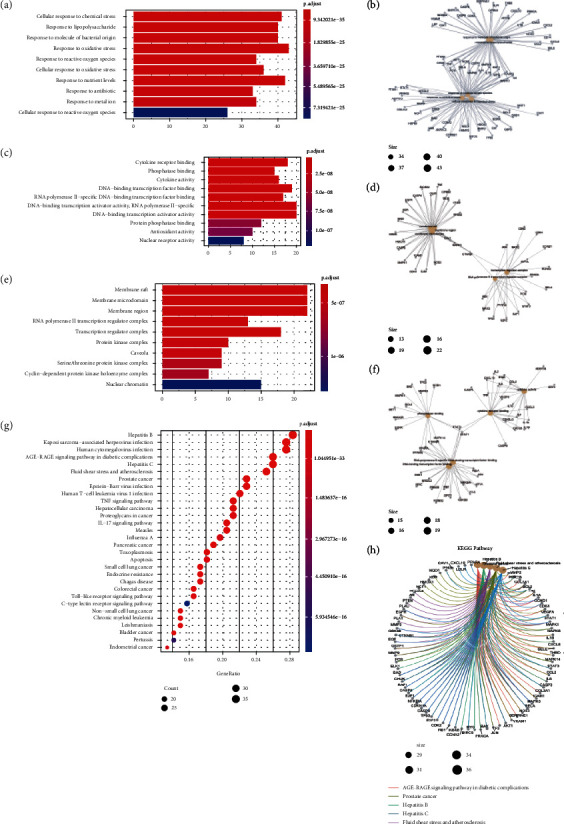
GO (including BP, MF, and CC) and KEGG analysis of common genes. (a) Top 10 significantly enriched terms in biological processes (BPs). (b) Subnetwork showing the top five BP terms and related genes. (c) Top 10 significantly enriched terms in cellular components (CCs). (d) Subnetwork showing the top five CC terms and related genes. (e) Top 10 significantly enriched terms in molecular functions (MFs). (f) Subnetwork showing the top five MF terms and related genes. (g) The 30 pathways with the lowest adjusted *p* values. The darker the color, the smaller the adjusted *p* value. The larger the circle, the greater the number of target genes in the term. (h) Subnetwork showing the top five KEGG pathways and related genes.

A total of 2617 items were acquired: 2417 from BPs, 60 from CCs, and 140 from MFs (Supplementary Table 4). The top 10 of each category are shown in Figure 5. The results showed that the biological processes of the potential genes are associated with oxidative stress, such as response to oxidative stress, response to reactive oxygen species, cellular response to oxidative stress, and cellular response to reactive oxygen species. The cellular component might be mainly activated in the membrane raft, membrane microdomain, membrane region, RNA polymerase II, transcription regulator complex, and transcription regulator complex. The molecular functions of common genes include cytokine receptor binding, phosphatase binding, cytokine activity, DNA binding, transcription factor binding, and RNA polymerase II-specific DNA-binding transcription factor binding.

**Figure 6:**
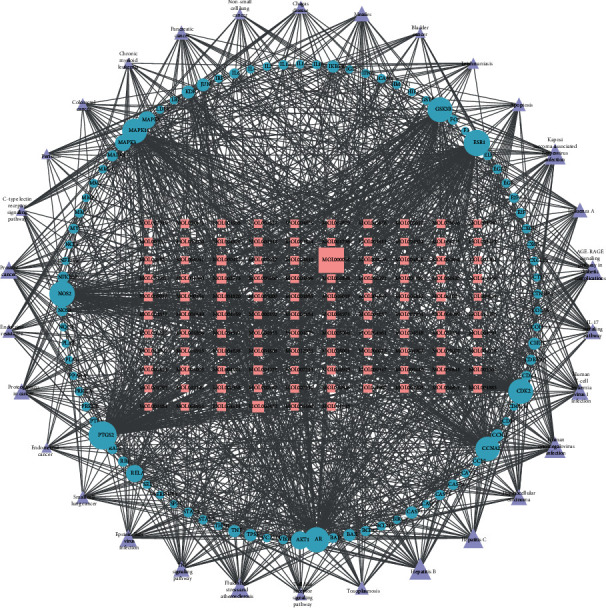
“Pathway-common target-active ingredient” network. Purple triangle nodes represent the top 30 IVDD-related pathways; blue circles represent common genes; pink rectangle represents the active ingredient of DSF.

Simultaneously, we obtained 166 KEGG pathways associated with potential genes (Supplement Table 4), of which the top 30 are shown in Figure 5. The meaningful pathways contain the AGE-RAGE signaling pathway involved in diabetic complications, the IL-17 signaling pathway, the TNF signaling pathway, the Toll-like receptor signaling pathway, and apoptosis. Although cellular senescence, the PI3K-Akt signaling pathway, and the FoxO signaling pathway are not shown in the diagram, they were also selected for further analysis because they may be related to inflammation, apoptosis, senescence, and autophagy. The results confirmed that DSF alleviated IVDD disease by regulating antioxidant stress and inflammatory reactions. The top 30 pathway-common target-active ingredient networks are shown in Figure 6.

### 3.5. Disease-Core Genes–Active Ingredients–Herbs

**Figure 7:**
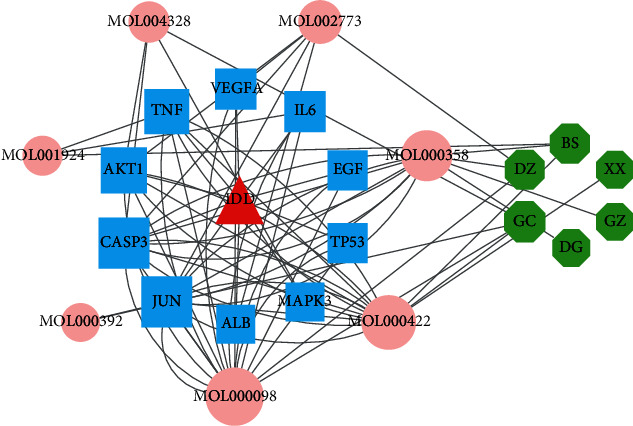
“IVDD-core gene–active ingredient–herb” network. Red triangle nodes represent disease, blue square nodes represent core genes, pink circle nodes represent the active ingredients related to the core genes, and the green diamond nodes represent herbs. The size of each node was set according to their degree value.

Based on the 10 core genes, we obtained seven active ingredients and six herbs. A network of disease core gene–active ingredient–herb interactions is shown in Figure 7 with 24 nodes and 70 edges. Quercetin (MOL000098) is the most crucial active ingredient with the largest degree, which is consistent with the results of the active ingredient-potential target network. JUN and CASP3 were the key genes with the highest degrees.

### 3.6. Molecular Docking

**Figure 8:**
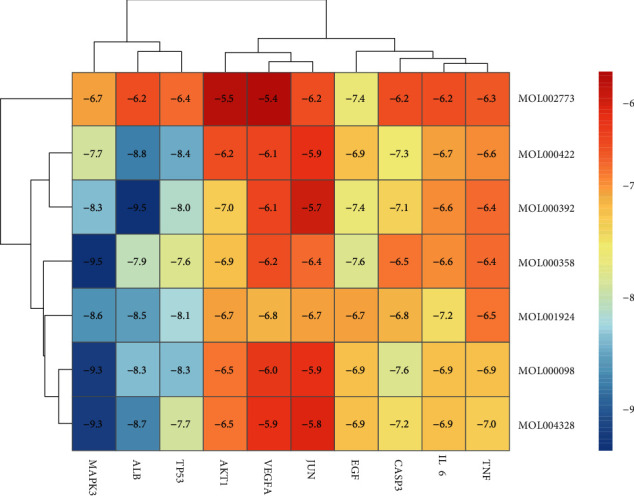
Heat map of molecular docking between 10 core genes and active ingredients of IVDD.

**Figure 9:**
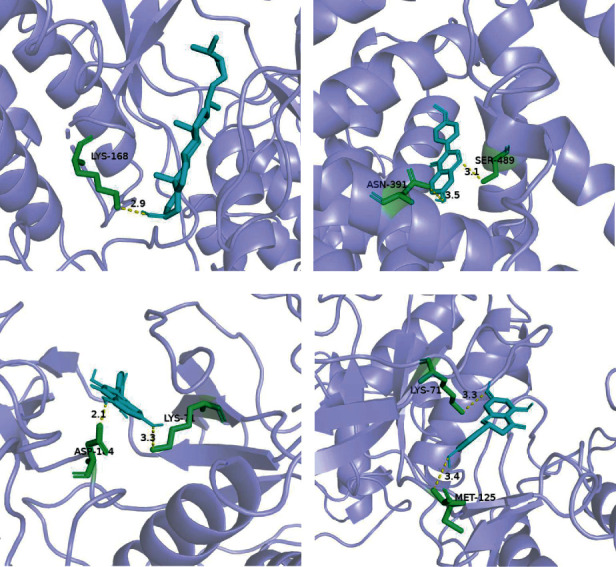
Molecular docking of the “bioactive compound-core gene”: (a) beta-sitosterol to MAPK3; (b) formononetin to ALB; (c) quercetin to MAPK3; (d) naringenin to MAPK3.

Ten key genes were selected for molecular docking with seven major active compounds to verify the binding ability between key active compounds and core gene interactions. Studies have shown that the lower the binding energy, the more stable the conformation of the compound binding to the protein, and the greater the possibility of interaction [26]. The docking results are shown in the heat map (Figures 8 and 9). It is generally believed that the binding energy between proteins and small molecule compounds is relatively stable when it is less than -5.0 kcal/mol [26]. As predicted by network pharmacology, our binding energy results were all less than -5.0 kcal/mol, indicating that all active compounds could easily enter the active pocket of the protein and bind stably. In addition, all compounds could bind to multiple targets simultaneously, suggesting the mechanism of the multitarget role of decoction in the treatment of intervertebral disc degeneration. In addition, all compounds can bind to multiple targets simultaneously, suggesting the mechanism of the multitarget role of DSF in the treatment of IVDD.

## 4. Discussion

IVDD is one of the common causes of low back pain. At present, the incidence of IVDD is increasing, which seriously aggravates patients' mental and financial burden [27, 28]. Previous evidence has shown that the occurrence and development of IVDD are related to inflammation and oxidative stress, leading to apoptosis and senescence of nucleus pulposus cells [29–32]. Drugs currently used to treat IVDD are limited to nonsteroidal anti-inflammatory drugs or muscle relaxants to relieve symptoms [1, 33]. Notably, TCM has been used for more than 2,000 years to treat various diseases, including IVDD [34]. Compared with Western medicine, TCM has the advantages of mild and fewer side effects. To date, network pharmacology has been widely used to study the mechanisms of TCM. In our study, the results of the active ingredient-potential target network showed that the main active compounds, including quercetin, beta-sitosterol, kaempferol, naringenin, and formononetin, may have potential research value for the treatment of IVDD. In addition, enrichment analysis showed that DSF acted on various biological processes of IVDD and influenced the disease course through eight pathways, such as the AGE-RAGE signaling pathway in diabetic complications, the IL-17 signaling pathway, the TNF signaling pathway, and the Toll-like receptor signaling pathway, which confirmed that DSF has multicomponent, multipathway, and multitarget characteristics.

Some of the main compounds in DSF identified in our study have anti-inflammatory and antioxidative stress effects, thereby inhibiting apoptosis and senescence. Quercetin and kaempferol are common ingredients in licorice. Previous studies suggested that quercetin has antisenescence and antiapoptotic effects, mainly by promoting SIRT1-dependent autophagy and inhibiting senescence-associated secreted phenotype factor expression and the senescence phenotype in nucleus pulposus cells to prevent and treat IVDD [35, 36]. In addition, Lu et al. found that quercetin could inhibit the expression and release of various inflammatory factors, such as TNF-*α*, IL-1*β*, and IL6, by suppressing the activation of the NF–*κ*B pathway [37]. Kaempferol has been confirmed to modify the osteogenesis/adipogenesis balance and inhibit inflammation in BMSCs, making it a new target for the treatment of IVDD [38]. The common component in Angelicae sinensis radix, cinnamomi, ramulus, Paeoniae radix alba, and Jujubae fructus is beta-sitosterol, which has anti-inflammatory and antioxidant effects [39]. Cho et al. found that formononetin may reduce the proteoglycan content used to treat musculoskeletal diseases [40].

By analyzing the PPI network of common genes, we found that AKT1, IL6, ALB, TNF, VEGFA, TP53, MAPK3, CASP3, JUN, and EGF might be the main potential targets for DSF therapy of IVDD. The apoptosis of nucleus pulposus cells (NPCs) plays a crucial role in the pathological process of IVDD, which is mainly mediated by inflammation and oxidative stress [3, 4]. IL6, TNF, TP53, and CASP3 are closely associated with the inflammatory response in the process of IVDD, among which TNF and IL6 are proinflammatory factors; moreover, TP53 and CASP3 induce apoptosis and senescence. AKT1 encodes one of the three members of the human AKT serine-threonine protein kinase family, which are often referred to as protein kinase B alpha, beta, and gamma. AKT/PI3K is a key component of many signaling pathways, and AKT proteins regulate a wide variety of cellular functions, including cell proliferation, survival, metabolism, and angiogenesis, in both normal and malignant cells. Moreover, Zhan et al. found that the degree of intervertebral disc degeneration was related to the loss of vascular buds and the downregulation of VEGFA and its receptors [41].

GO and KEGG enrichment analysis results showed that the therapeutic targets of DSF for diseases mainly focused on the AGE-RAGE signaling pathway involved in diabetic complications, IL-17 signaling pathway, TNF signaling pathway, Toll-like receptor signaling pathway, apoptosis, cellular senescence, PI3K-Akt signaling pathway, and FoxO signaling pathway. The biological processes are mainly induced in response to oxidative stress and reactive oxygen species and the regulation of apoptotic signaling pathways. Studies have shown that all of these factors play crucial roles in the progression of IVDD. The TNF and IL-17 pathways play a synergistic role in the progression of IVDD, mainly by promoting the release of inflammatory factors, the apoptosis of NPCs, and the degradation of extracellular matrix (ECM) [42–44]. mTOR is a serine/threonine protein kinase activated by PI3K/Akt, ERK, Wnt, TNF-*α*, IGF1 or low energy, low oxygen, and other factors. Then, mTOR activates downstream 4E-BP1 and p70S6K, which play a vital role in regulating the proliferation, apoptosis, and nutritional status of interdisc cells [45]. Furthermore, activation of the PI3K-Akt pathway may lead to a series of events, including reduction of ECM degradation, inhibition of apoptosis, and induction or suppression of autophagy, which can protect against IVDD [46, 47]. Recent studies have shown that FOXO is a critical regulator of cellular homeostasis during IVDD [48]. In addition, Gao et al. found that 17*β*-estradiol prevents ECM degradation by downregulating MMP3 expression via the PI3K/Akt/FOXO3 pathway [49]. At present, among the regulatory pathways of IVDD, few studies on the correlation between IVDD and other signaling pathways were screened out in our study by KEGG analysis. A study found that accumulation of AGE-MG-H1 was associated with endochondral ossifications, hypertrophy, and osteogenic differentiation in IVDD and that these are directly related to RAGE, suggesting that AGE/RAGE could be a potential therapeutic target [50].

However, our study also has some limitations. For example, the results of this study lacked in vitro validation, and further external validation with animals should be performed. Moreover, the screening of active ingredients and related genes through databases may not be sufficiently comprehensive.

## 5. Conclusions

The combination of network pharmacology and molecular docking provides a practical way to analyze the molecular biological mechanisms of DSF-mediated IVDD treatment. AKT1, IL6, ALB, TNF, VEGFA, TP53, MAPK3, CASP3, JUN, and EGF may be potential targets of DSF in treating IVDD, and enrichment analysis results showed that the therapeutic targets of DSF for diseases mainly focused on the AGE-RAGE signaling pathway in diabetic complications, IL-17 signaling pathway, TNF signaling pathway, Toll-like receptor signaling pathway, apoptosis, cellular senescence, PI3K-Akt signaling pathway, and FoxO signaling pathway, which confirms the multicomponent, multipathway, and multitarget characteristics of DSF and provides an essential theoretical basis for clinical practice.

## Figures and Tables

**Table 1:** Full scientific species names of herbs of Danggui-Sini formula.

Pin Yin	Latin name
Dang Gui (DG)	Angelica sinensis
Gui Zhi (GZ)	Cinnamomum cassia Presl
Bai Shao (BS)	Cynanchum otophyllum Schneid
Xi Xin (XX)	Asarum sieboldii Miq
Gan Cao (GC)	Glycyrrhiza uralensis Fisch
Tong Cao (TC)	Tetrapanax papyriferus
Dao Zao (DZ)	Ziziphus jujuba Mill

**Table 2:** Cluster information of the protein-protein interaction (PPI) network for common genes.

Cluster	Score	Nodes	Edges	Gene symbol
1	48.483	59	1406	IL10, MAPK1, NFKBIA, MAPK14, CXCL10, MMP1, CASP9, CASP8, STAT1, CD40LG, PLAU, RELA, CDKN1A, HIF1A, CCND1, *MAPK3*^∗^, BCL2L1, AR, STAT3, *CASP3*^∗^, KDR, *VEGFA*^∗^, *ALB*^∗^, *TP53*^∗^, SPP1, CXCL8, PPARG, *JUN*^∗^, EGFR, PTEN, ESR1, MYC, *EGF*^∗^, *AKT1*^∗^, SIRT1, ADIPOQ, MPO, CAT, IL1B, *TNF*^∗^, FOS, PTGS2, *IL6*^∗^, HMOX1, MMP2, SERPINE1, CCL2, IL2, IFNG, IL4, CRP, ICAM1, VCAM1, NOS3, MMP3, NOS2, SELE, MMP9, MAPK8

2	4.706	18	40	IGF2, SOD1, CXCL2, NFE2L2, IL1A, IRF1, HSPB1, CCNB1, CDK4, CDK2, RB1, CCNA2, NCF1, GSK3B, CAV1, GJA1, CTNNB1, RUNX2

3	3	3	3	PLAT, THBD, F3

4	3	3	3	NRI12, CYP3A4, CYP2B6

5	2.5	5	5	G6PD, HMGCR, FASN, ACHE, PON1

^∗^Core genes are shown in italic.

## Data Availability

The data used and analyzed during the current study was available from the corresponding author on reasonable request.

## Abbreviations

IVDD: Intervertebral disk degeneration
NPCs: Nucleus pulposus cells
NSAIDs: Nonsteroidal anti-inflammatory drugs
TCM: Traditional Chinese medicine
DSF: Danggui-Sini formula
TCMSP: Traditional Chinese Medicine Systems Pharmacology
OB: Oral bioavailability
DL: Drug likeness
OMIM: Online Mendelian Inheritance in Man
CTD: Comparative Toxicogenomics Database
GO: Gene ontology
KEGG: Kyoto Encyclopedia of Genes and Genomes
BPs: Biological processes
CCs: Cell components
MFs: Molecular functions
PPI: Protein-protein interaction
NPCs: Nucleus pulposus cells
ECM: Extracellular matrix.

## Supplementary Materials

Supplementary Materials Supplementary Table 1: detail information for active ingredients of Danggui-Sini formula. Supplementary Table 2: potential targets related to active ingredients. Supplementary Table 3: Common targets related to intervertebral disc degeneration. Supplementary Table 4: the results of GO and KEGG enrichment analysis.
