# Supplementary figures and images for: Pharmacological Mechanism of Danggui-Sini Formula for Intervertebral Disc Degeneration: A Network Pharmacology Study
Source: Biomed Res Int. 2021 Nov 11;2021:5165075. doi: 10.1155/2021/5165075 (PMC8601842; doi:10.1155/2021/5165075)

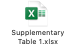
 Please double-click to view the table.

Supplement: Supplementary Materials — Supplementary Table 1: detail information for active ingredients of Danggui-Sini formula. Supplementary Table 2: potential targets related to active ingredients. Supplementary Table 3: Common targets related to intervertebral disc degeneration. Supplementary Table 4: the results of GO and KEGG enrichment analysis. [file 5165075.f1.zip › Supplementary Table 1.docx]

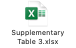
 Please double-click to view the table.

Supplement: Supplementary Materials — Supplementary Table 1: detail information for active ingredients of Danggui-Sini formula. Supplementary Table 2: potential targets related to active ingredients. Supplementary Table 3: Common targets related to intervertebral disc degeneration. Supplementary Table 4: the results of GO and KEGG enrichment analysis. [file 5165075.f1.zip › Supplementary Table 3.docx]

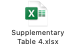
 Please double-click to view the table.

Supplement: Supplementary Materials — Supplementary Table 1: detail information for active ingredients of Danggui-Sini formula. Supplementary Table 2: potential targets related to active ingredients. Supplementary Table 3: Common targets related to intervertebral disc degeneration. Supplementary Table 4: the results of GO and KEGG enrichment analysis. [file 5165075.f1.zip › Supplementary Table 4.docx]
